# Interventions to improve tolerability of local anesthetic intradetrusor Botulinum toxin injections: A systematic review

**DOI:** 10.1002/nau.25061

**Published:** 2022-10-23

**Authors:** Nicholas Faure Walker, Finn Macpherson, Ali Tasleem, Tarannum Rampal

**Affiliations:** ^1^ King's College Hospital NHS Foundation Trust London UK; ^2^ School of Immunology & Microbial Sciences Faculty of Life Sciences and Medicine, King's College London London UK; ^3^ Faculty of Life Sciences and Medicine, King's College London King's College Hospital NHS Foundation Trust London UK; ^4^ King's College Hospital NHS Foundation Trust & Greenwich University London UK

**Keywords:** Botulinum toxin, local anesthetic, overactive bladder, tolerability

## Abstract

**Introduction:**

Intradetrusor BotulinumtoxinA (BTA) injections are recommended for patients with overactive bladder (OAB) refractory to lifestyle changes and medical intervention. It is preferable to perform injections using a flexible cystoscope under local anesthetic (LA) rather than under spinal or general anesthetic owing to the associated anesthetic risks, increased costs, and need for repeated inpatient admission. Injections under LA can be difficult to tolerate for some patients. This review aims to assess interventions that may improve the tolerability of intradetrusor BTA injections under LA.

**Methods:**

A systematic review was performed using Ovid of Embase + Embase classic and MEDLINE® ALL in November 2021. Articles were included if they reported objectively measured pain scores during LA intradetrusor BTA injections for refractory OAB. The risk of bias was assessed using Cochrane risk of bias tools. Meta‐analysis was not performed owing to the heterogeneity of outcome measures.

**Results:**

Ten studies were included in this review with a total of 429 participants. The review identified alkalinized lidocaine, electromotive drug administration (EMDA), opiate suppositories, lidocaine bladder instillations, number of injections, and dose of BTA as interventions aimed at improving tolerability.

**Conclusion:**

EMDA of intravesical alkalinized lidocaine, intravesical, alkalinized lidocaine without EMDA, and a reduction in the number of injection site were all associated with improvements in patient tolerability during LA BTA injections. Further research should address which subgroups of patients find the procedure most painful and would benefit most from these interventions.

## 1 INTRODUCTION

Overactive bladder (OAB) is defined by the International Continence Society as urgency, with or without urge incontinence, usually with frequency and nocturia.^1^ First‐line treatment involves conservative lifestyle measures before medical treatment with oral antimuscarinic or Beta 3 agonist medication.^2^ However, many patients either cannot tolerate the side effects of medication or their symptoms are not sufficiently controlled.^3^, ^4^, ^5^ Intradetrusor Botulinum toxin A (BTA) injections have been shown to be very effective in the management of refractory OAB in patients^6^, ^7^, ^8^, ^9^ and the procedure is recommended as first‐line treatment for refractory OAB in both idiopathic and neuropathic patients by NICE and EAU.^2^, ^10^ The procedure can be performed either under local or general anesthetic (GA). Performing the procedure under local anesthetic (LA) is more desirable as not only is the patient not subject to the associated risks of repeated GAs, but it is also significantly more cost effective keeping in mind that patients often have to return for repeat injections, typically every 9 months for patients with idiopathic OAB and 6 months for patients with neuropathic lower urinary tract dysfunction (NLUTD).^11^ Furthermore, reducing time in hospital during the current COVID‐19 pandemic is particularly favorable. Some studies have reported patient tolerability and have found that the procedure is generally well tolerated.^12^, ^13^, ^14^, ^15^, ^16^, ^17^, ^18^ Nevertheless, many patients find this procedure painful and cannot tolerate it under LA. The aim of this review is to evaluate interventions which may improve the tolerability of LA intradetrusor BTA injections and avoid the need for spinal anesthetic or GA.

## 2 MATERIALS AND METHODS

This systematic review was undertaken using standard methods recommended by the Preferred Reporting Items for Systematic reviews and Meta‐Analyses (PRISMA) guidelines.^19^ The review was registered with PROSPERO (registration number CRD42022311533).

### 2.1 Search strategy

An electronic search was performed using Ovid of Embase + Embase classic and MEDLINE® ALL in November 2021 for articles reporting patient tolerability and pain outcomes for intradetrusor botox injections for OAB. Search terms included “[botox OR botulinum toxin] AND [detrusor OR intra‐detrusor OR intradetrusor] AND [pain OR tolerability OR anaesthetic].” Two reviewers screened abstracts and titles together. Any disagreements were resolved by discussion.

### 2.2 Inclusion criteria

Studies evaluating intradetrusor BTA injections in adults (>18 years) for patients with refractory OAB that reported tolerability in the form of objective pain scores for the procedure were included. Studies that included information on drop‐out rates were not included if they did not report objective measures of pain. Non‐English language studies, literature reviews, nonprospective studies, and guidelines were excluded. Conference abstracts were included if relevant.

### 2.3 Outcome measures

The primary outcome measure was the tolerability of intradetrusor BTA injections in the form of pain scores using a visual analogue score (VAS) or similar numerical or spatial scale.

### 2.4 Data extraction and synthesis

Number of participants and intervention were extracted from each study along with data on VAS taken during the procedure, with and without intervention. Data were analyzed according to intervention to improve the tolerability of LA intradetrusor Botulinum toxin injection.

### 2.5 Risk of bias

The risk of bias was assessed using the Cochrane risk of bias 2.0 tool for randomized controlled trials and the Cochrane ROBINS‐I tool for nonrandomized studies.

## 3 RESULTS

### 3.1 General study characteristics

**Figure 1:**
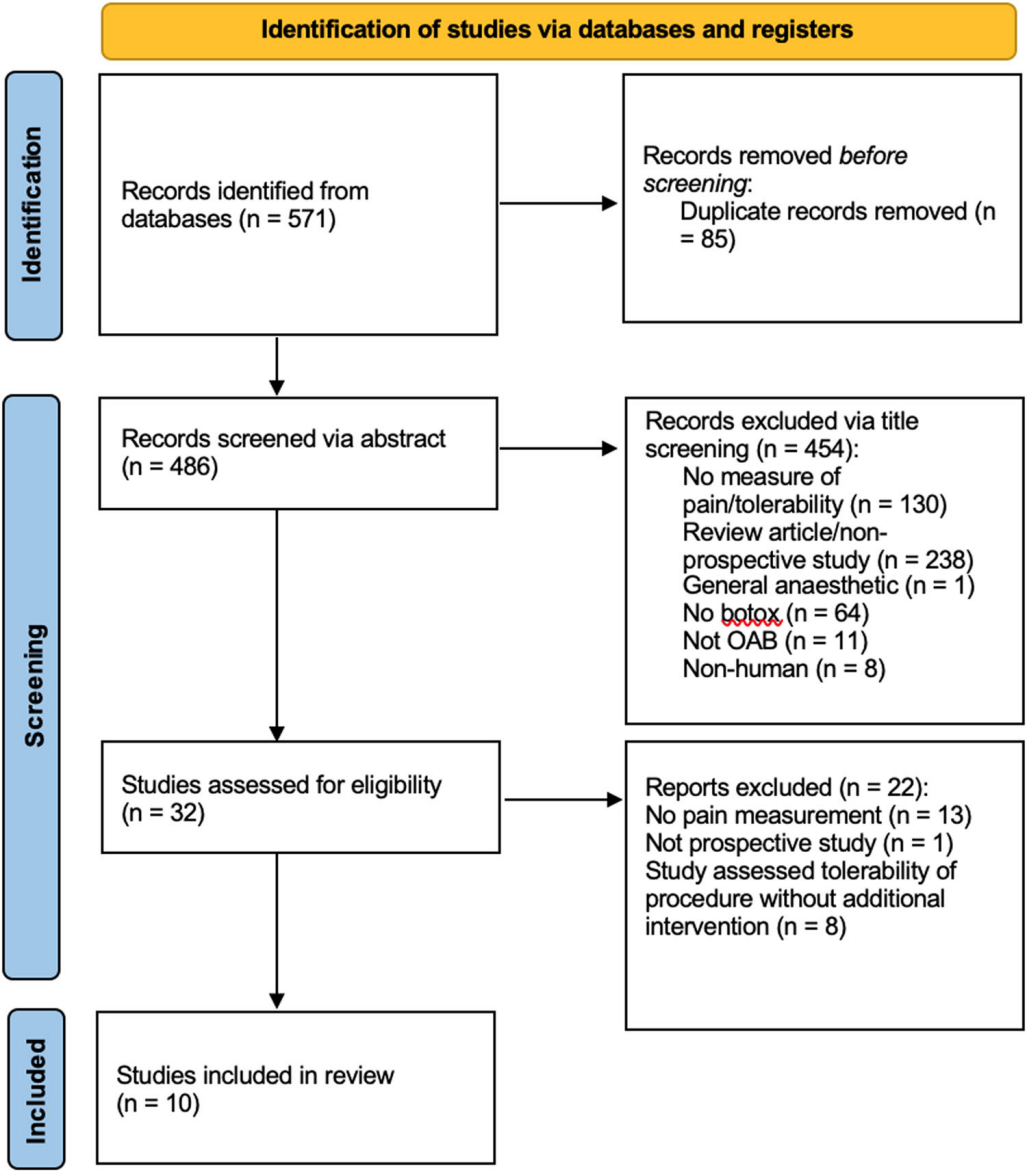
Preferred Reporting Items for Systematic reviews and Meta‐Analyses flow diagram

Overall, 10 studies were included in the final analysis. The study selection process is shown in Figure 1. Study characteristics were tabulated and presented in Table 1 which summarises the studies, including the nature of the study, the number of participants, the specific intervention, and a summary of outcomes. Any eligible prospective trials were included whether a full text was available or not. The measure of tolerability across all studies was a VAS pain score or similar.

**Table 1:** Studies included in this systematic review

References	Study design	Number of participants	Intervention	Summary of findings
Briskin et al.^20^	Prospective study	25	Number of injection sites	Number of injection sites showed weakly negative correlation with pain scores
Cohen et al.^21^	Prospective study	27	Number of injection sites	Nonsignificant reduction in pain with fewer injections
Denys et al.^22^	Double‐blind randomized controlled trial	99	Range of doses	No significant difference in pain with different doses of BoNTA
Du et al.^23^	Single‐blind randomized controlled trial	14	Anesthetic method—intravesical versus gel	No statistically significant difference
Kocher et al.^24^	Prospective, nonrandomized study	25	Lidocaine anesthetic protocols + alkalinized lidocaine	No statistical difference in pain scores
LeClaire et al.^25^	Randomized double‐blind placebo‐controlled study	26	Opiate analgesia	No significant difference in pain scores with opiate suppository
Lo and Lo^26^	Prospective randomized study	40	Number of injection sites	Reduction in postoperative pain with fewer injections
Pedraza Sanchez et al.^27^	Prospective study	29	Intraurethral lidocaine gel versus intravesical alkalinized lidocaine	No statistically significant difference in pain scores
Pereira e Silva et al.^28^	Double‐blind randomized controlled trial	116	Alkalinised lidocaine	Alkalinised lidocaine superior to lidocaine only
Schurch et al.^29^	Prospective, open‐label, cross‐over‐designed study	28	Electromotive drug administration (EMDA)	EMDA enhancement significantly reduced pain scores

### 3.2 Anesthesia

#### 3.2.1 Lidocaine bladder instillations

Du et al. compared 20 ml 1% lidocaine intravesical instillation + 10 ml aquagel via the urethra with 0.9% normal saline solution instillation with 10 ml 2% lidocaine gel via theurethra.^23^ They found there to be no statistically significant difference between pain scores on a 100 mm VAS scale between urethral lidocaine gel and intravesical lidocaine instillation. The study group had a median pain score of 15 (interquartile range [IQR]: 10–43.3) compared to 22.5 (IQR: 13.8–56.9) in the control group using gel alone.

#### 3.2.2 Alkalinized lidocaine

The studies by Pereira e Silva et al.,^28^ Kocher et al.,^24^ and Pedraza Sanchez et al.^27^ evaluated the effectiveness of lidocaine alkalinized with bicarbonate solution.

Pereira e Silva et al. performed a double‐blinded, randomized controlled trial comparing intravesical instillations of 20 ml 2% lidocaine + 10 ml 8.4% sodium bicarbonate with 20 ml 2% lidocaine + 10 ml 0.9% saline solution immediately before BTA injections. Both groups received urethral lubrication gel. The trial included a total of 116 patients, of which 100 (86.2%) were female, 85 (73.3%) had idiopathic detrusor overactivity (IDO), 21(18.1%) had neurogenic detrusor overactivity (NDO) and 10 (8.6%) were being treated for bladder pain syndrome (BPS). The subjects who had received alkalinized lidocaine solution reported lower pain scores immediately after the procedure than those who received lidocaine solution with saline (NRS 2.37 ± 0.31 and 4.44 ± 0.36, respectively, *p* < 0.01). There was no difference in perceived pain one hour after the procedure.

Kocher et al.^24^ compared different lidocaine instillations including alkalinized lidocaine (100 ml of 1% or 2% lidocaine and 50 ml 2% lidocaine + 10 ml bicarbonate) and the influence this had on pain in a prospective study involving 25 patients. There was no statistically significant change in patient‐reported discomfort using VAS for different lidocaine instillations (*p* = 0.913), nor for dwell time of the instillation (*p* = 0.14).

Pedraza Sanchez et al.^27^ also compared alkalinized lidocaine instillation with intraurethral lidocaine gel as part of a prospective study which included 29 women. They too found no significant difference in VAS between those who received an intravesical alkalinized lidocaine instillation and those who received intraurethral lidocaine gel.

#### 3.2.3 Electromotive drug administration (EMDA)

Schurch et al.^29^ carried out an open‐label, cross‐over study which looked at the effectiveness of intravesical lidocaine with and without EMDA in patients with neurogenic lower urinary tract dysfunction. A majority of the patients (24/28 = 85.7%) had suffered spinal cord injury and two patients (7.1%) had myelomeningocele. There were 10 patients in the conventional (non‐EMDA) group who received 40 ml 2% lidocaine solution, which was drained after 20 min using a Foley catheter. When these patients’ incontinence recurred, they were converted to the EMDA group with 18 other patients. In the EMDA group, 75 ml of 4% lidocaine was mixed with 75 ml of sterile water and 1.5 ml 1/100 000 epinephrine. The urethra was also lubricated with 20 ml 2% lidocaine gel. The bladder was washed out with 100 ml saline before 150 ml of the lidocaine solution was instilled. The negative electrodes were placed on the abdominal skin. The pulse current was activated and increased progressively (40–60 mA/s to a maximum of 25 mA, for 20–25 min, total charge of 600 mA). The mean VAS in the group who underwent EMDA was 0.7, which was significantly less than the conventionally administered lidocaine group (mean VAS 4). Patients in the EMDA group who had undergone the procedure before without EMDA also stated that they would prefer EMDA‐enhanced lidocaine for future BTA injections. The authors also analyzed costs assuming the EMDA generator could provide 100 treatments. EMDA‐enhanced instillation was found to cost CHF 491 compared to CNF 582 for spinal or GA.

#### 3.2.4 Addition of opiate suppository

In a randomized controlled trial of rectal belladonna and opium (B&O) suppository versus placebo in 26 idiopathic, female participants, LeClaire et al.^25^ assessed pain VAS before anesthesia, 40 min after anesthesia, after the first 10 BTA injections, and after 20 injections. The median VAS after 10 and 20 injections was 5 and 2 for the B&O group and 4 and 3 for the placebo group. Any difference was not statistically significant.

### 3.3 Number of injections

The number of injection sites varied throughout all the studies. Three studies looked at how the number of injection sites affected tolerability.^20^, ^21^, ^26^ Lo and Lo^26^ found that 10 sites resulted in a significantly reduced postoperative pain (35%) compared to 20 sites.

Briskin and Atiemo^20^ also assessed VAS in patients undergoing 12–22 injections. They found a weakly negative correlation with increasing VAS, *r* = −0.191 (−0.545, 0.221). This study also demonstrated a weakly positive correlation with increasing anesthetic dwell time, *r* = 0.362 (−0.038, 0.663) and age, *r* = 0.333 (−0.072, 0.643). Mean VAS was also found to be higher in idiopathic patients, 5.4 (3.86, 6.94) compared to neuropathic, 4.3 (2.87, 5.73).

Cohen et al.^21^ compared 100 U (10 sites) with 150 U (15 sites) and although patients with 10 sites experienced less pain (VAS = 2.3) than those with 15 (VAS = 3.5), the difference was not statistically significant (*p* = 0.28).

### 3.4 BTA dose

Denys et al.^22^ was the only study identified comparing VAS in patients undergoing different doses of BTA but using the same number of injection sites. They found the peri‐surgical VAS “at around 4 out of 10” for all groups including the placebo (placebo = 4.0 ± 2.6, BTA: 50 U = 3.9 ± 2.4, 100 and 150 U both = 4.2 ± 2.5).

### 3.5 Risk of bias

**Figure 2:**
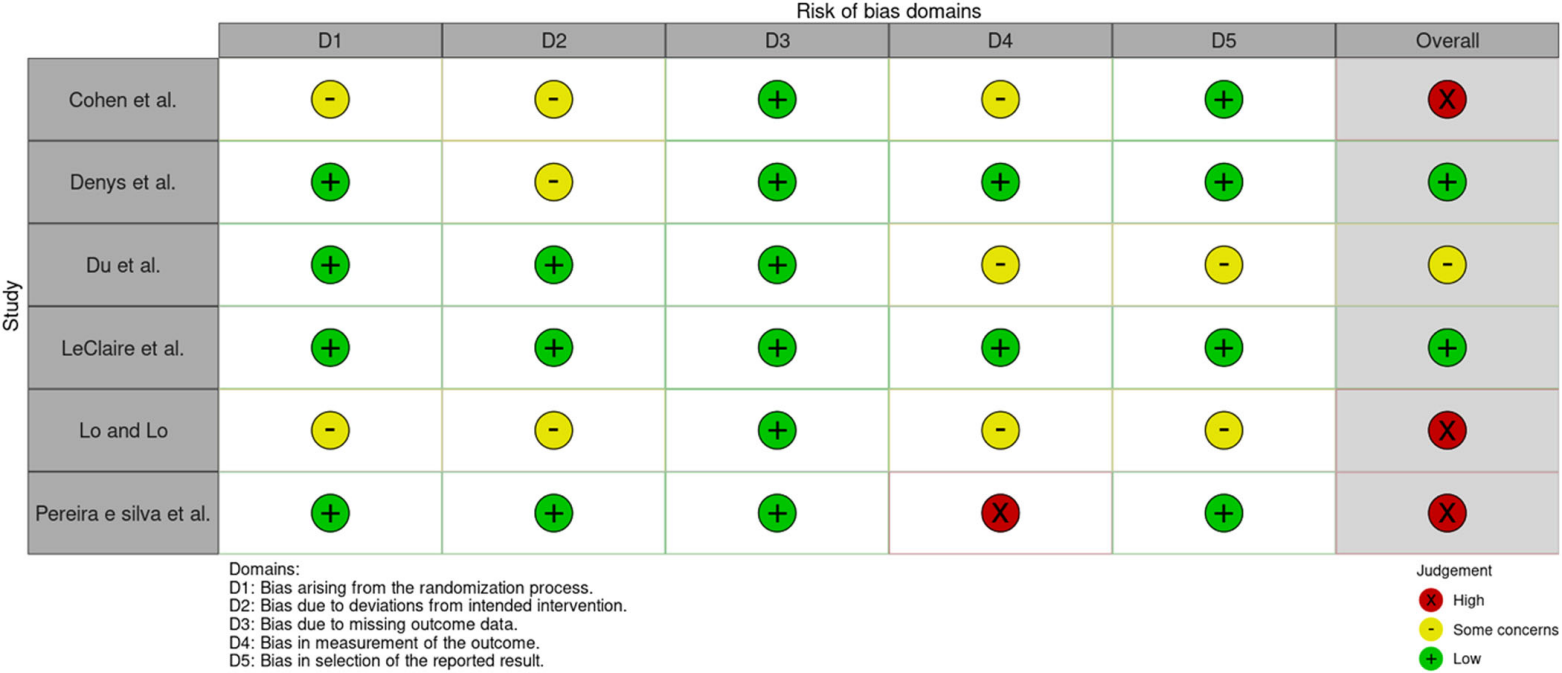
Risk of bias assessment for randomized controlled trials

**Figure 3:**
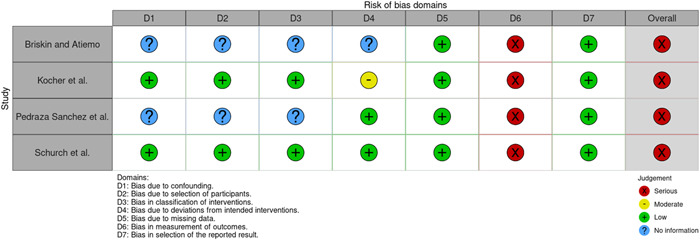
Risk of bias assessment for nonrandomized studies

A summary of the assessment is presented in Figures 2 and 3.

## 4 DISCUSSION

This review aimed to identify and assess interventions that could potentially improve the tolerability of LA intradetrusor BTA injections. BTA injections typically need to be repeated every 6 months for patients with neurogenic lower urinary tract dysfunction and every 9 months for patients with idiopathic refractory OAB. It is hence paramount to ensure that LA intradetrusor BTA injections are tolerable under LA to avoid the risks and financial implications of repeated general or spinal anesthetic.

Intravesical lidocaine was assessed in several studies. When used with lubricating urethral gel (without lidocaine), intravesical (non‐alkalinized) lidocaine was not shown to be of any benefit compared to placebo in a randomized trial.^23^ Three studies, including one randomized trial, compared intravesical, alkalinized lidocaine with intraurethral lidocaine gel anesthesia.^24^, ^27^, ^28^ While Pedraza Sanchez et al. showed some improvement in pain score immediately after the procedure, there was no difference in pain scores an hour later. The other studies did not show any improvement in pain scores with alkalinized lidocaine. It is not clear whether there is any benefit of an intravesical installation of alkalinized lidocaine solution in addition to intraurethral lidocaine gel. Furthermore, another study which looked at the effectiveness of intravesical, alkalinized lidocaine found that the alkalinized lidocaine solution cost twice that of the intraurethral lidocaine gel.^30^ The most profound improvement in pain scores was observed in Schurch et al.'s^29^ study which evaluated the effectiveness of electromotive administration (EMDA) of alkalinized lidocaine. Intravesical lidocaine with EMDA reduced mean pain scores from 4.0 to 0.5. The subjects were all neuropaths. A reduction in the number of injections appeared to reduce pain levels in two studies. Rectal opiate analgesia was not shown to improve pain scores compared to placebo. Changes in dose also did not affect perceived pain levels.

Most LA including lidocaine are weak bases and are not completely ionized at physiological pH. They, therefore, cross the phospholipid neuronal membrane more easily in their unionized state,^31^ which is why they are less effective in infected, acidic tissues. The acidic environment of the bladder may also explain why non‐alkalinized, intravesical lidocaine was not shown to reduce pain scores in patients undergoing LA BTA injections.

The sensory neurons of the bladder comprise of myelinated Aδ fibers and unmyelinated c fibres.^32^ The Aδ fibers are found within the detrusor muscle and respond to stretch. The c fiber nociceptors are responsible for conveying pain and are found within the detrusor muscle, the lamina propria, and in the submucosal layer of the bladder.^33^ Via iontophoresis (the movement of ions down a concentration gradient), electrophoresis (the electrically induced convective flow of water with ions across a coulombic gradient) and electroporation (increasing membrane permeability using an electric field), EMDA has been shown to improve the intravesical penetration of drugs such as Mitomycin (MMC) used in the treatment of superficial bladder cancer.^34^ MMC is a non‐ionized, weaky polarised molecule. Sodium ions flow by iontophoresis along their charge gradient and the MMC molecules follow via electro‐osmosis thus increasing the depth of penetration into the bladder wall tissues. The use of EMDA‐driven MMC sequentially with Bacillus Calmette–Guerin (BCG) has been shown to result in a 50% progression‐free survival at 2 years in patients refractory to BCG alone. It is likely that by driving lidocaine deeper into the bladder wall using EMDA, Schurch et al. greatly improved the tolerability of LA BTA injections by allowing the lidocaine to act upon the c fibers, which lie within the deeper layers of the bladder as well as those within the mucosa. Schurch et al. also stated that EMDA alkalinized lidocaine does not require specialist training to administer and the costs were calculated to be approximately 16% less than general or spinal anesthesia.

This review identified three studies that identified that reducing the number of injections reduced pain scores.^20^, ^21^, ^26^ The sample size was however small for all three studies (range 25–40). Two of these studies also assessed whether fewer injections were associated with a reduction in effectiveness. Cohen et al.^21^ reported excellent efficacy with decreased doses of 100 or 150 U BTA at 10 or 15 injection sites, respectively, compared to the traditionally used 20 or 30 sites (200 or 300 U), while Lo and Lo^26^ also noted no difference in efficacy between those receiving 10 injections compared with those receiving 20 injections. The DIGNITY study (Double‐Blind Investigation of Purified Neurotoxin Complex in Neurogenic Detrusor Overactivity) was the first significant randomized trial of intradetrusor BTA in patients with NLUTD. The injection technique in the DIGNITY study involved 30 injection sites, 1 cm apart.^7^ The technique from this pivotal RCT has been widely replicated ever since though it would appear^30^ that a significant number of practitioners now deviate from the recommended number of injection sites in the United States. A small number of studies have since assessed how reducing the number of injection sites affects the outcomes of BTA injections. Reinjection with as few as three or four sites has been shown to be equally effective as the standard technique of 30, hence a reduction in injection sites may represent a simple way of improving the tolerability of LA BTA without compromising effectiveness.^35^

Of studies that assessed tolerability in the outpatient setting without intervention, all reported the procedure to be generally well tolerated. However, there was no standardized measurement for what constituted tolerability. Pain is a subjective experience and influenced by a multitude of factors.^28^ Myles et al.^36^ found 33 mm on a 100 mm VAS to be the upper limit of acceptable pain for postoperative patients. Perception of the event was considered by Ballert and Nitti^16^ and Chang et al.,^17^ albeit from different perspectives. The former identified that patients found the procedure to be less painful than anticipated whereas the latter found the procedure to be more painful than professionals performing the procedure believed it would be for the patients. This highlights a potential disconnect between the patient and the healthcare professional when considering the tolerability of a procedure.

Most of the identified studies reported mean scores which may not encapsulate the small number of outlying patients who experienced very severe pain. These would be the key patients to identify as they are the ones who may require spinal or GA. The effect of patient factors including age, sex, and underlying pathology on tolerability was not well explored in the identified literature. Specific pain studies in the wider literature have identified that women are more sensitive to pain than men on most pain sensitivity measures. Age effects of pain sensitivity are relatively weak.^37^ Within the literature identified in this systematic review, Briskin and Atiemo^20^ was the only study to look at specific patient subgroups. They found VAS to be lower among neurogenic patients compared with that of idiopathic OAB patients. The majority of the studies were small and had a predominantly female cohort. There were also heterogenous mixes of idiopathic and neuropathic patients. Consequently, larger studies identifying which subgroups of patients tolerate the procedure most poorly would be valuable as these patients would benefit the most from the interventions identified in this review. Reitz et al.^38^ found a notable increase in bladder sensation and pain perception in patients who exhibited bladder cold sensation when tested with ice water instillation. Understanding which patients experience bladder cold sensation may allow the prediction of lower tolerability as well. Although it would be more cumbersome to assess this for every patient than simply having a prediction based on a subgroup study, it is an example of how identifying relevant subgroups can be informative of the likelihood of experiencing more pain.

Heterogeneity across studies was high, and the lack of concordance and standardization of protocol across different centers reduced direct comparability. Many of the studies measuring tolerability were doing so as a secondary outcome and used different methods of administration and anesthesia. Bias was also high throughout, partially influenced by the lack of full texts available and the nature of patient‐reported pain scores as an outcome.

## 5 CONCLUSION

LA BTA injections for refractory OAB are generally well tolerated in studies which report pain scores. EMDA of alkalinized lidocaine appears to be the most effective intervention in reducing pain during LA intradetrusor BTA injections. There may be a modest improvement in tolerability with intravesical, alkalinized lidocaine without EMDA. A reduction in the number of injection sites was also shown to improve tolerability. These interventions may help patients avoid the need for repeated spinal or GA injections. Intravesical lidocaine solution without alkalinization, dose reduction, and rectal opiate analgesia were not found to improve pain scores. Further research should address which subgroups of patients find the procedure most painful and would benefit the most from these interventions.

## AUTHOR CONTRIBUTIONS

**Nicholas Faure Walker**: Concept, data collection, supervision, and writing manuscript. **Finn Macpherson**: Data collection and drafting manuscript. **Ali Tasleem**: Writing and review of manuscript. **Tarannum Rampal**: Review of manuscript.

## CONFLICT OF INTEREST

The authors declare no conflict of interest.

## Data Availability

This is a systematic review. Data is readily available from the internet.
